# Rehabilitation Living Lab in the Mall Community of Practice: Learning Together to Improve Rehabilitation, Participation and Social Inclusion for People Living with Disabilities

**DOI:** 10.3390/ijerph120404439

**Published:** 2015-04-22

**Authors:** Barbara Mazer, Dahlia Kairy, Andréanne Guindon, Michel Girard, Bonnie Swaine, Eva Kehayia, Delphine Labbé

**Affiliations:** 1 Centre for Interdisciplinary Research in Rehabilitation of Greater Montreal, Jewish Rehabilitation Hospital, 3205 Place Alton Goldbloom, Laval, Quebec H7V 1R2, Canada; E-Mail: eva.kehayia@mcgill.ca; 2 School of Physical and Occupational Therapy, McGill University, 3654 Promenade Sir William Osler, Montreal, Quebec H3G 1Y5, Canada; 3 Centre for Interdisciplinary Research in Rehabilitation of Greater Montreal, Gingras-Lindsay Rehabilitation Institute of Montreal, 6300 Darlington Avenue, Montreal, Quebec H3S 2J4, Canada; E-Mail: dahlia.kairy@umontreal.ca; 4 School of Rehabilitation, University of Montreal, 7077 Parc Avenue, Montreal, Quebec H3N 1X7, Canada; E-Mail: bonnie.swaine@umontreal.ca; 5 Centre for Interdisciplinary Research in Rehabilitation of Greater Montreal, Constance-Lethbridge Rehabilitation Centre, 7005 De Maisonneuve O. Boulevard, Montreal, Quebec H4B 1T3, Canada; E-Mail: a.guindon.clethb@ssss.gouv.qc.ca; 6 Collaboration Achieved by Communities of Practice and Networks; E-Mail: michel@rezomichelgirard.com; 7 Centre for Interdisciplinary Research in Rehabilitation of Greater Montreal, Lucie-Bruneau Rehabilitation Centre , 2275 Laurier East, Montreal, Quebec, H2H 2N8, Canada; E-Mail: psi.coordinator@gmail.com

**Keywords:** community of practice, knowledge sharing, knowledge building, collaboration, disability, social participation

## Abstract

Communities of practice (CoP) can facilitate collaboration between people who share a common interest, but do not usually work together. A CoP was initiated and developed including stakeholders from clinical, research, community and governmental backgrounds involved in a large multidisciplinary and multi-sectorial project: the Rehabilitation Living Lab in a Mall (RehabMaLL). This study aimed to evaluate the structure, process and outcomes of this CoP. A single case-study, using mixed-methods, evaluated the RehabMaLL CoP initiative after one year, based on Donabedian’s conceptual evaluation model. Forty-three participants took part in the RehabMaLL CoP with 60.5% (n = 26) participating at least once on the online platform where 234 comments were posted. Four in-person meetings were held. Members expressed satisfaction regarding the opportunity to share knowledge with people from diverse backgrounds and the usefulness of the CoP for the RehabMaLL project. Collaboration led to concrete outcomes, such as a sensitization activity and a research project. Common challenges included lack of time and difficulty finding common objectives. A CoP can be a useful strategy to facilitate knowledge sharing on disability issues. Future research is necessary to determine strategies of increasing knowledge creation between members.

## 1. Introduction

Researchers in the field of rehabilitation are increasingly interested in finding mechanisms to ensure their research is relevant and applicable for front-line health care professionals and community partners, with the ultimate goal of increasing social participation of people with disabilities. Models describing the complex process of bringing different types of knowledge into action, such as the Knowledge-To-Action (KTA) model [1], highlight the necessity to include stakeholders early in the research process in order to develop projects relevant to their needs and realities [2]. This model implicitly presents knowledge transfer and knowledge application as a two-way process, taking into account both knowledge empirically derived (research) as well as other experiential or tacit knowledge such as that gained from clinical practice or personal experiences.

Despite this growing interest in including relevant stakeholders in the KTA process, difficulties in involving them in research remain. This gap between the research being conducted and the needs and realities of stakeholders, has been identified for different groups, including clinical practitioners [3,4,5], community organizations [6] and policy decision-makers [7,8]. Creating spaces to promote collaborative, sense-making and improvised conversation between different stakeholders in which learning can take place is essential to reducing this gap [9]. A vast array of collaborative research approaches have been proposed to increase the implementation of research findings into practice [2,10,11,12]. The premise is that by bringing together individuals from research and clinical domains early in the process, these groups will better understand each other, thus making research findings more useable for the clinicians [10,13]. Kothari *et al.*, [14] have thus argued that there is a need for a much wider range of stakeholders in public health-based KTA processes.

Communities of Practice (CoP) have been presented as a KTA strategy that can facilitate recognizing and supporting the use of different types of knowledge and evidence, such as tacit or explicit knowledge from a variety of stakeholders [14,15]. A CoP is an efficient and potentially effective approach to forging collaboration between different groups of people. CoPs can be defined as: “groups of people who share a concern, a set of problems, or a passion about a topic, and who deepen their knowledge and expertise in this area by interacting on an ongoing basis” [16] (p.4). They can serve different purposes, including sharing ideas, solving problems, disseminating best practices or organizing knowledge [16]. Advantages of collaborating in a CoP include: greater sharing of knowledge; reduced isolation; building competencies; and facilitating collaboration that can lead to innovation [17,18,19]. Some authors suggest that CoPs allow research findings not only to be transmitted, but to be contextualized so that they may become more applicable for the different stakeholders [20,21]. CoPs can also allow more timely access to new research findings [21]*.* As well, through the ongoing discussions that take place in a CoP, participants can build up a stock of knowledge and resources from which they can draw when needed [22]. Grol and Grimshaw [23] suggest that CoPs can bridge the gap between what is current research and practice. 

There seems to be an increasing interest in using CoPs in general [24], and in health care in particular [18,20]. In the healthcare sector, CoPs commonly seek to foster sharing and learning between members, but are diverse in their composition, number of participants, mandate and communication tools [18,20,25], as well as in the assessment framework used for their evaluation [25,26]. Promising outcomes have been documented for CoPs seeking to facilitate the KTA process [27,28,29], as well as for those working to bridge the gap between research and stakeholders [15,17,20,21,30,31]. However, there is little evidence on how to successfully develop and implement a CoP to facilitate collaboration between different stakeholders in the community interested in a common topic, such as disability and social participation. The objective of this study was to evaluate the ongoing RehabMaLL CoP (structure, process and outcome) and to examine the participants’ perspectives regarding their experience of collaborating in the CoP on social participation over the first 20 months. 

## 2. Methods 

### 2.1. Study Design

A single case-study, triangulating both qualitative and quantitative data, was used to evaluate the RehabMaLL CoP initiative. 

### 2.2. RehabMALL Community of Practice and Study Participants

A CoP was initiated in May 2013 to facilitate mutual sharing of ideas and experiences between stakeholders from research, clinical, community and governmental milieus involved in the Rehabilitation Living Lab in the Mall (RehabMaLL; www.crir-livinglabvivant.com), a large multidisciplinary and multi-sectorial research project initiated by the Centre for Interdisciplinary Research in Rehabilitation of Greater Montreal (CRIR). The RehabMaLL has established partnership with an international real-estate promoter and owner of a Montreal downtown shopping mall, local community groups, as well as local, national and international research, clinical and industry partners. The RehabMaLL is the first environment of its kind, designed to study how to best address the needs of persons of all ages with physical, sensory and cognitive disabilities. It aims to promote their social participation by creating an inclusive mall environment where numerous interactions and daily activities typically take place. Due to the complexity of this project and the multiple stakeholders involved, a strategy was needed to facilitate collaboration between all partners. The CoP was thus created to implement, support and structure knowledge creation and translation, to best ensure the progress of the RehabMaLL project. Its development and accompanying evaluation was led by two researchers, (Barbara Mazer, Dahlia Kairy), from the RehabMaLL. 

The CoP sought to recruit participants from different backgrounds, including rehabilitation clinicians and managers from the clinical establishments, researchers participating in the RehabMaLL project, representatives of community organizations and advocacy groups, people with disabilities and their family members as well as representatives from the partnering shopping mall in Montreal, Canada. For community stakeholders, including people with disabilities, and community and governmental organizations, recruitment was done by word of mouth and personal recommendations from the RehabMaLL project managers. All interested individuals were invited to participate, regardless of their previous level of involvement in the RehabMaLL project. Clinicians were recruited from the clinical sites and all those who manifested an interest and met the inclusion criteria were included. In order to ensure a representation of researchers from diverse fields, these participants were invited based on recommendations from the RehabMaLL project managers and through the CRIR research center. Inclusion criteria for all participants included interest and expertise in the area of social participation of people living with disabilities, openness to change and desire to share with others, comfort with the fact that both English and French languages would be used in the CoP, and access to a computer and the internet. All members of the CoP consented to participate in the accompanying evaluation process.

### 2.3. Data Sources and Collection 

There are a limited number of frameworks [25,26] and validated tools available to address the relevant constructs potentially impacted by the CoP. The use of multiple data sources to evaluate CoPs has been recommended in the literature [18]. 

In order to assess and evaluate the CoP, Donabedian’s conceptual evaluation model for quality of care [32] was selected and adapted (Table 1). The model defines quality of care as a combination of three elements: *structure* (the characteristics of the setting of care, such as material and human resources and organizational structure), *process* (what is actually done by the different actors) and *outcome* (the effects of care on patients and population). Notions of quality and performance are directly linked to the organizational structure of the health system and to its management of processes. The outcomes correspond to the quality of care offered to the patients and to the performance of the health system in offering this care. 

**Table 1:** Donabedian’s model adapted for evaluating the community of practice (CoP).

Structure	Process	Outcome
Characteristics of the members of the CoP and types of participants Characteristics of the platform being used Structure of the CoP meetings and online communication *Sources of data*: logs, interviews	Usage patterns and vitality of exchanges between participants Changes in the CoP involvement (change in user numbers, vitality, functions used) Type of information and knowledge shared *Sources of data:* logs, web data, questionnaires, interviews	Member satisfaction Achievement of individual and group objectives Concrete results and outcomes Changes in the participants and organizations (in knowledge or practice) *Sources of data*: logs, web data, questionnaires, interviews

In this study, data from the online platform were collected over the first 15 months, from May 2013 to July 2014. Following completion of this first portion of the analysis, administration of questionnaires and telephone interviews with the participants was conducted from August to December 2014. A log and notes from the in-person CoP meetings were maintained throughout. 

#### 2.3.1. Questionnaires 

*Questionnaire on communities of practice*. The CEFRIO, a research network interested in the use of technologies to foster innovation, developed a questionnaire specifically for examining different facets of CoPs. It was developed in the context of a study where multiple CoPs in different sectors were initiated and evaluated [33,34]. The 40 items addressed in the questionnaire are derived from a comprehensive search of possible factors that can impact on the success of a CoP. They are divided in the following sections: results (e.g., “I think the overall objectives of the CoP have been achieved”), learning experiences (e.g., “In the CoP, I learned a great deal on a personal level”), value of the initiative (e.g., “The CoP is very useful for the organization that sponsored it”), vitality of exchanges (e.g., “Members ask questions in order to gain a better understanding”), relationships between members (e.g., “There is a strong sense of belonging to the CoP”), obstacles to collaboration (e.g., “My participation in the CoP meetings requires too much time”), outcomes (e.g., “The CoP allows for an exchange of information with people working in the same field as I do”) and member satisfaction (e.g., “I am very satisfied with my participation in the CoP”). Participants are asked to rate their agreement with each statement on a scale of 1 to 5 (1 = strongly disagree to 5 = strongly agree). In light of the diversity in types of participants and in their level of involvement in the RehabMaLL CoP, the options “I don’t know” and “Not applicable” were also added. The original French version was translated to English by a professional, and validated by two experts in rehabilitation and knowledge translation research. It was pre-tested for clarity of questions with two persons not participating in the RehabMaLL CoP. Participants completed the questionnaire in the language of their choice. 

*Newman’s survey for online communities*. Four additional questions based on a questionnaire developed by Newman *et al.* [35] were also used to evaluate participants’ satisfaction with the CoP online platform (e.g., The online platform is reliable). The original questionnaire by Newman *et al.* had been developed to evaluate an online conference and was based on Salmon’s [36] five stage model for developing online communities. The questions address reliability, access, ease of use and visual appearance of the platform. 

#### 2.3.2. Web Data

Data from the online platform were collected over the first 15 months of the CoP and included: number of discussion topics and comments, number of comments made by each participant and broad content of discussions. 

#### 2.3.3. Phone Interviews

Interviews with CoP participants were conducted in French or English by two independent interviewers familiar with the RehabMaLL project, but who had not been part of the CoP activities. Interviews included six open-ended questions for researchers and seven for other participants. Themes addressed were: positive and negative aspects of collaborating using the online platform and during in-person meetings, changes in perspective on research and usefulness of the CoP for research activities, as well as impact of the CoP on their own knowledge and activities. The seventh question addressed participant’s (other than researchers) interest to take part in future research activities following their experience in the RehabMaLL CoP. Participants took part in the phone interview at a time convenient to them and all interviews were recorded. 

#### 2.3.4. Logs and Meeting Summaries 

The CoP facilitator kept a log throughout the first year of the RehabMaLL in order to collect data on the original structure of the RehabMaLL and changes to the structure over time, as well as the activities of the CoP and regular meetings between the facilitator and the main researchers (Barbara Mazer, Dahlia Kairy), who insured the governance of the project. Summaries of meetings were also produced and shared with CoP members, by email and on the online platform. Participants who attended could give feedback on the exactitude of the notes, and they helped those who had been absent keep up-to-date with the activities.

### 2.4. Data Analysis

A summary of the data collected, using the structure, process and outcome dimensions of Donabedian’s model, as applied to the CoP, is presented in Table 1. All members were attributed a participant code (C-clinician, CO-community organization, R-researcher, CP-community participant, G-representatives of governmental agencies, F-facilitator or O-other), followed by a number. 

#### 2.4.1. Questionnaires

Answers were regrouped (disagree: 1–2, neutral: 3, agree: 4–5) in order to allow us to identify trends. Descriptive analysis using frequencies were conducted per item, for each individual and for categories of participants. A Fisher’s Exact Test compared scores between categories of participants. To control for multiple comparisons, a *p* < 0.01 level of significance was used. All analyses were done using SAS Software. 

#### 2.4.2. Web Data 

Information regarding each entry on the web platform was compiled by a research assistant (*i.e.*, topic name, participant code, date of comment, and the participant’s comment). Comments were then categorized into themes according to its content. Some themes were also subdivided. Comments may have been included in more than one theme. This process was validated by the research team (Barbara Mazer, Dahlia Kairy and Andreanne Guindon). Themes were chosen based on a classification proposed by Connected Educators, a group interested in innovations seeking to foster knowledge sharing using social media and online connections (http://connectededucators.org: Resources and Tools for Evaluation of Online Communities of Practice). They include: Practice, Resource, Advice, Collaboration, Novel Ideas, Summary, Vulnerability, Asking, and Appreciation (Table 2). After the categorization was complete, descriptive analyses were conducted.

**Table 2:** Description of themes and subthemes for online data.

Theme	Description of Themes and Subthemes
Practice	Information regarding the job and tasks they do or do not do or would like to do. *Setting* *or background of practice*: (a) clinical, (b) research, (c) organization, (d) in person’s life, (e) in the CoP
Resources	Posts or documents that share specific resources. *Type of resource* : (a) website/ application, (b) document, (c) video *Content:* (a) community resources, (b) health resources (rehabilitation, medical), (c) awareness, (d) researcher
Advice	Posts in relation to a situation or a problem expressed by another participant that suggests solutions or propositions or opinions.
Collaboration	Posts that contain offers of collaboration/introduction to potential collaborators. *Precision on collaboration*: (a) Posts where participants offer to collaborate on a specific activity or task (b) Posts where participants offer their organization’s collaboration with something. (c) Posts where participants ask for collaboration.
Novel Ideas	Posts that reply to a post by suggesting a new idea in relation to a subject/problem, that is not an existing resource or that is not only the explanation of a lived experience.
Summary	Posts that contain a summary of previous posts or meetings.
Vulnerability	Posts in which the person admits not knowing something / having a difficulty.
Asking	Post in which a person asks for something. *Type of information asked about* : (a) advice/opinion, (b) help/support, (c) clarification
Appreciation	Posts which express appreciation or agreement.

#### 2.4.3. Interviews 

Interviews were first summarized to identify key points addressed for each question. A content analysis of the interviews was then conducted. Any discrepancies were discussed by the research team. In total, 369 ideas were coded into 25 categories. 

### 2.5. Ethical Considerations

All subjects gave their informed consent for inclusion before participating in the study. The study was conducted in accordance with the Declaration of Helsinki, and the protocol was approved by the Ethics Committee of the Center for Interdisciplinary Research in Rehabilitation of Greater Montreal (CRIR-796-0113). 

## 3. Results

Results from the quantitative and qualitative data are presented in the following section according to the dimensions of Donabedian’s model: structure, process and outcomes. 

The number of participants in the CoP varied over time due to the nature of the group which has ongoing recruitment and involvement that can depend on the participant’s interest in the topic of discussion or activity. Forty-three participants were included in the online data collection and 28 participants took part in the questionnaire and interviews (Table 3). 

**Table 3:** Total number of participants for each aspect of the study.

Number of Participants at Each Stage of the Study				
Invited to take part in the CoP	Agreed to participate	Participated online	Participated in at least one meeting	Answered questionnaire and open-ended interview
50	43	26	41	28

### 3.1. Findings Related to Structure of the CoP 

Elements considered to inform about the structure of the CoP include characteristics of the members and of the platform being used, as well as the structure of the online discussions, meetings and activities. Data sources include facilitator’s log and interviews. 

Fifty people were contacted to take part in the CoP, of which 43 participated at some point, including researchers in rehabilitation, clinicians, members of community organizations, people living with a disability and family members, as well as representatives of governmental agencies (Table 4). The majority of participants were female (72%). There were no participants recruited from industry and commercial partners of the RehabMaLL project, despite repeated efforts. 

The RehabMaLL CoP was designed to evolve through in person meetings, as well as through an online collaborative platform, with the support of a facilitator. The online platform was offered to facilitate ongoing exchanges between members regardless of availability and location of the participant. Following an in-depth review of available online platforms, the platform chosen for this CoP was created by the Canadian Network for Public Health Intelligence (www.cnphi-rcrsp.ca). This platform has been in development for over 10 years to facilitate sharing of information between healthcare professionals throughout Canada. It includes a news board, discussion forum, library, list of members and an event calendar. Each CoP member had a username and password to access the platform. As a strategy to engage participation, monthly newsletters and summaries of meetings were sent out through the online platform and by email. Two short online surveys were also conducted using the online platform to determine participants’ interests and to help organize CoP activities. 

**Table 4:** Description of participants who accepted to take part in the RehabMaLL CoP.

Participant Type	Examples of Participants’ Backgrounds	Total Number of Participants
Facilitator and co-facilitator	RehabMaLL facilitator and consultant for the CoP	2
Clinician	Occupational therapist, psychologist, speech and language therapist mostly from rehabilitation centers and community settings	13
Community Organization	Advocacy groups, resource centers, organizations offering information about leisure and/or tourism for people living with disabilities	8
Community Participant	People living with physical disabilities and a family member	3
Representative of Governmental Agency	Government representative from the Ministry of Health and Social Services of Quebec, the Office for Disabled People of Quebec, municipal council	4
Researcher	Researchers from the Center for Interdisciplinary Research in Rehabilitation of Greater Montreal	7
Other	RehabMaLL project manager, representative from a Quebec museum, representative of another living lab with people living with disabilities, research assistant	6
Total		43

### 3.2. Findings Related to Process of the CoP 

Elements considered to inform us about the process of the CoP include vitality of exchanges, changes in participation over time and type of knowledge shared. Data presented comes from the facilitator’s log, web data, questionnaires and open-ended questions. 

Despite the original estimate of two yearly meetings, due to the dynamic discussion that occurred during these events, four in-person meetings were held from June 2013 to December 2014. Participation in each of the meetings ranged from 19 to 24 participants, in addition to the presence of the facilitator, a consultant for the CoP (also acting as co-facilitator; Michel Girard), the main researchers for the CoP (Barbara Mazer, Dahlia Kairy) and the scientific directors of the RehabMaLL (Eva Kehayia, Bonnie Swaine). All but two (out of the 43) members participated in at least one of the in-person meetings. Participants in the in-person meetings were involved in research presentations, small working groups, discussions, and planning of community activities.

Online participation was sporadic in most cases. Of the 43 participants who took part in the CoP activities over the first 15 months, 60% were active on the online platform at least once (Table 5). On average, participants posted 5.4 comments, with facilitators contributing the majority (56.5). When facilitators were removed, the average number of comments per participant was 3.0 and members of community organizations and clinicians were the most active contributors. The majority of the discussions were initiated by the facilitator and co-facilitator (69.5%). 

**Table 5:** Total number of comments online over the first 15 months according to participant type.

Participant Type	Total Number of Participants	Total Number of Participants Online	Total Number of Comments (Mean per Participant)	Number of Discussions Initiated
Facilitator and co-facilitator	2	2	113 (56.5)	25
Clinician	13	8	37 (2.9)	3
Community Organization	8	6	39 (4.9)	4
Community Participant	3	3	6 (2)	0
Government	4	1	5 (1.3)	0
Researcher	7	3	21 (3)	2
Other	6	3	13 (2.2)	2
Total	43	26	234 (5.4)	36

**Figure 1:**
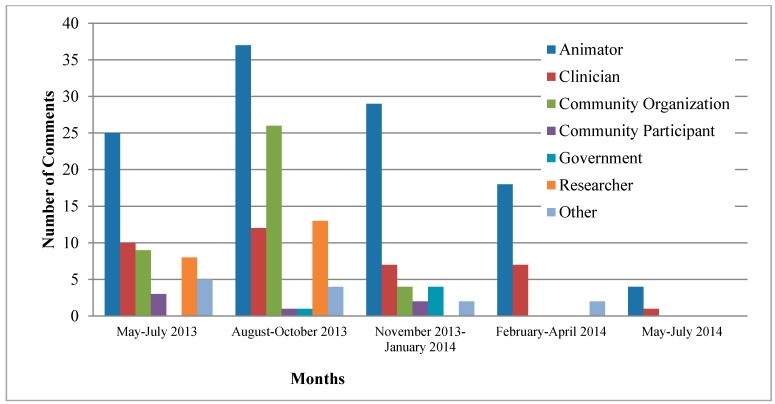
Number of comments over time according to type of participant.

The number of monthly comments did not display a visible pattern, with peaks in July (47), October (54) and November (33), 2013. An important decline can be observed after a few months following the creation of the CoP (Figure 1). 

**Figure 2:**
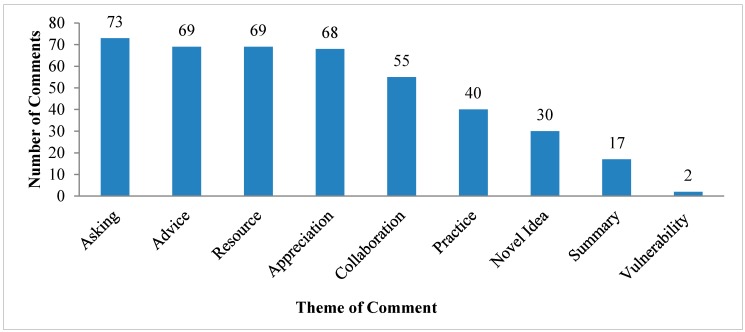
Total number of comments posted online by theme.

Figure 2 presents the categorization of comments posted online into the nine predetermined themes. Numbers include all comments by participants and facilitators. Asking a question to other members was the most common category, followed by posts suggesting a solution or proposition to someone else’s question, identifying specific resources, and expressing appreciation or agreement. Comments also sought or proposed potential collaborations, which is one of the goals of the RehabMaLL CoP. 

Different types of participants tended to post about certain themes. Aside from vulnerability, the facilitators were the most frequent contributors for each theme. When facilitators were removed, clinicians were most likely to give advice (n = 16) and discuss practice (n = 14), while community organizations most often expressed appreciation (n = 16), asked questions (n = 11) and shared resources (n = 11). 

Examples of topics addressed online are: the use of public places like the shopping mall in the rehabilitation process, phone applications to evaluate the accessibility of public places, accessible tourism and leisure, and ways of sensitizing storeowners about accessibility. The most popular topics and the ones that could be linked to research in the RehabMaLL were discussed more in-depth during the meetings. 

Results from the questionnaires administered to CoP members are presented in Table 6. Sections of the questionnaire referring to the process of the CoP, such as vitality of exchanges, relationships, obstacles and perception of the online platform are addressed here. Sections referring to the outcomes (marked with * in Table 6) are addressed in the following section. Participants agreed that other members asked each other questions to get a better understanding (100%), explained their ideas (96%), made positive comments at appropriate times (88.4%), and did not hesitate to raise a new subject in order to advance the work (88.4%). Participants all agree that CoP members have something to offer to each other (100%), and that even if members have personal objectives, they all share a common goal (87%). Lowest scores obtained were related to the online platform. Participants were satisfied with its reliability (87%), but much less with the ease of use (53.8%) or visual appearance (41.7%). There were no significant differences between types of participant. 

Data collected during the interviews also provided insight into the process of the CoP. In particular, questions addressed the preferred mode of collaboration of members, either online or in-person, as well as positive or negative aspects of both. Participants who had used the online platform reported some advantages regarding the platform as compared to in-person meetings, including the possibility to discuss and share resources on an ongoing basis, as well as it being a central place to keep track of information. Difficulty accessing and using the platform, either because of its design or from lack of familiarity with technology, was mentioned as an obstacle. Limited participation of members online was also perceived as an issue, making discussions less dynamic than they would have liked. Finding the time to go online is the most common obstacle expressed by participants during the interviews. Questionnaire results show that 29.6% of participants agree that their participation in the CoP requires too much time. 

In-person meetings, on the other hand, facilitated discussion and sharing with the other members, according to participants’ interviews. It allowed for dynamic and stimulating exchanges, leading to new reflections and increased awareness of different perspective. The meetings were also the occasion to network and forge collaborations. However, some participants reported that it was sometimes hard to find a focus to the meetings or specific discussions that would be relevant to everyone. 

There was a clear preference for collaborating during the in-person meetings, rather than online, although a few participants noted that if exchanges were dynamic on the on-line platform, it would be best to communicate both in-person and on-line. 

Based on the questionnaires, few obstacles to participating in the CoP received high scores. Results of the interviews indicate that time is the main factor limiting participation in the CoP. 

### 3.3. Findings Related to Outcome of the CoP 

Elements considered to inform us about the outcomes of the CoP include member satisfaction, achievement of personal and group objectives, development of new initiatives, as well as change in individuals and organizations. Data sources include questionnaires, open-ended interviews, logs and minutes of meetings. 

Both online and during the in-person meetings, the diversity of the members and the opportunity to share different points of view on a variety of relevant issues were much appreciated, as expressed during the open-ended interviews. Results from the questionnaires show that 100% of participants agree that members have something to offer each other. Overall, 55.6% of participants reported satisfaction with their own participation in the CoP.

Participants highly agree that the CoP allows them to exchange information with people working in the same field as them (92.3%), to speak about their own experiences (88.5%) and to see the advantage of working together with others in similar organizations (88%). With regards to learning from the CoP, members agree they learned on a personal (77%) and on a professional (70%) level. However, only 66% agree that it has helped them reduce professional isolation. Open-ended questions indicated that exchanges in the CoP had led some participants to reflect on new ideas or projects, and that the CoP was a good place to share information.

In relation to research activities, 100% of members agree that the CoP is useful for the RehabMaLL project (100%), and that the CoP has achieved its goal (87.5%). Open-ended questions reveal that researchers and participants in the category “other” also highly value the contributions of the RehabMaLL CoP for validating their research results and building collaborations. Some of them recommend working in smaller groups on targeted topics. For other members (community organization, clinician, community participant and representatives of government agencies), the RehabMaLL was an opportunity to gain a better understanding about research, to build collaborations and gain access to researchers. In some cases, the RehabMaLL CoP has been an incentive to participate in upcoming research activities. There were no significant differences between type of participant on any items from the questionnaire. 

**Table 6:** Results from the questionnaires on communities of practice (CEFRIO) and the online platform (Newman).

Category	Question		General (n = 28)		
		Number of respondants	n (%)		
			Disagree	Neutral	Agree
* Results	I think the overall objectives of the community of practice (CoP) have been achieved.	24	2 (8.3)	1 (4.2)	21 (87.5)
* Learning	In the CoP, I learned a great deal on a professional level.	25	3 (12)	4 (16)	18 (72)
	In the CoP, I learned a great deal on a personal level	26	3 (11.5)	3 (11.5)	20 (77)
	My skills for teamwork or for working in a CoP have grown	26	4 (15.4)	4 (15.4)	18 (69.2)
* Value	The CoP is very useful for the organization that sponsored it, which, in this case, is the MALL project	22	0 (0)	0 (0)	22 (100)
	The CoP is very useful for my employer	19	5 (26.3)	4 (21.1)	10 (52.6)
**Vitality of Exchanges**	Members share information easily	26	3 (11.5)	2 (7.7)	21 (80.8)
	Members ask questions in order to gain a better understanding	26	2 (7.7)	1 (3.9)	23 (88.4)
	Members easily give explanations to the others	25	0 (0)	1 (4)	24 (96)
	Members provide examples of what they are suggesting	24	0 (0)	0 (0)	24 (100)
	Members do not hesitate to raise a new subject in order to advance the work	26	2 (7.7)	1 (3.9)	23 (88.4)
	Members show good humor when appropriate	25	0 (0)	6 (24)	19 (76)
	Members make positive comments when appropriate	27	1 (3.7)	1 (3.7)	25 (92.6)
	Some members monopolize the discussion	27	16 (59.3)	6 (22.2)	5 (18.5)
	Some members are too critical	26	21 (80.8)	3 (11.5)	2 (7.7)
	Some members are too competitive	26	20 (76.9)	4 (15.4)	2 (7.7)
	Some members are too defensive	25	22 (88)	3 (12)	0 (0)
	Some members are too sarcastic	25	21 (84)	3 (12)	1 (4)
**Relationships**	There is a strong sense of belonging to the CoP	24	6 (25)	2 (8.3)	16 (66.6)
	The members share a common goal	26	1 (3.9)	4 (15.4)	21 (80.7)
	Each individual has personal objectives, but they are close to those of the CoP	23	0 (0)	3 (13)	20 (87)
	Members have something to offer me	27	0(0)	0 (0)	27 (100)
	Each individual has personal objectives that differ from those of the CoP	22	6 (27.3)	9 (40.9)	7 (31.8)
	The sharing of information has increased over the months	20	4 (20)	4 (20)	12 (60)
	Closeness among the members has increased over the months	20	4 (20)	2 (10)	14 (70)
	Cohesion among the members has increased over the months	21	2 (9.5)	5 (23.8)	14 (66.7)
**Obstacles to Collaboration**	I have trouble trusting the members of the CoP	25	23 (92)	1 (4)	1 (4)
	My participation in the CoP meetings requires too much time	27	13 (48.2)	6 (22.2)	8 (29.6)
	The current culture in my organization does not promote knowledge sharing	25	21 (84)	1 (4)	3 (12)
	I do not really see the advantages of working in a CoP	27	22 (81.4)	3 (11.1)	2 (7.4)
	The financial benefits of working in a CoP are impossible to measure	19	5 (26.3)	4 (21.1)	10 (52.6)
	Working in a CoP requires skills that I do not have	26	20 (77)	3 (11.5)	3 (11.5)
	I hesitate to share my professional expertise in the CoP because I am worried that it will have a negative impact on my organization	20	17 (85)	1 (5)	2 (10)
	The fact that there are members of the CoP who belong to organizations that are my competitors stands in the way of exchanges	18	15 (83.3)	1 (5.6)	2 (11.1)
* Outcomes	The exchanges within the CoP allow you to avoid isolation	24	4 (16.7)	4 (16.7)	16 (66.6)
	The CoP allows you to speak about your own experiences	26	1 (3.9)	2 (7.7)	23 (88.5)
	The CoP gives members the chance to hear concrete reports from people who are experiencing the same challenges as me	24	3 (12.5)	2 (8.3)	19 (79.2)
	The CoP allows for an exchange of information with people working in the same field as I do	26	2 (7.7)	0 (0)	24 (92.3)
	My participation in the CoP allows me to see the advantage of working together with others in similar organizations	25	2 (8)	1 (4)	22 (88)
	My participation in the CoP allows me to reduce the level of professional isolation	26	6 (23.1)	3 (11.5)	17 (65.4)
* Member Satisfaction	I am very satisfied with my participation in the CoP	27	8 (29.6)	4 (14.8)	15 (55.6)
	I would be interested in continuing to participate in a CoP	26	3 (11.5)	2 (7.7)	20 (76.9)
	Overall, my participation in the CoP has increased my satisfaction at work	24	6 (25)	3 (12.5)	15 (62.5)
* Member Satisfaction	I find my participation in the CoP to be very enriching on a personal level	23	3 (13)	5 (21.7)	15 (65.3)
	I find my participation in the CoP to be very enriching on a professional level	25	3 (12)	2 (8)	20 (80)
**Online Platform**	The online platform is reliable	23	0 (0)	3 (13)	20 (87)
	It is easy to access the platform	26	5 (19.2)	5 (19.2)	16 (61.6)
	The online platform is easy to use	26	8 (30.8)	4 (15.4)	14 (53.8)
	The online platform is visually appealing	24	9 (37.5)	5 (20.8)	10 (41.7)

* Sections related to outcome will be presented in the following section.

Collaboration within the RehabMaLL CoP has also led to new projects such as a sensitization activity regarding accessibility. This activity was organized by RehabMaLL CoP members, in collaboration with a Montreal community organization, also a collaborator in the RehabMaLL CoP. It corresponded to one of the goals that participants had mentioned throughout the meetings to help sensitize storeowners and workers to disability and universal accessibility. During the activity, teams including people with and without disabilities, whether members of the CoP or not, visited 54 stores to rate a variety of accessibility facets, such as the doors, washrooms, counters, *etc.* according to their own perspectives. In many cases, the participants engaged discussion with store owners and workers. 

The CoP participants also contributed to a specific research project led by one of the members, who is a clinician and a graduate student. The project received funding to survey current clinical practices and needs regarding the use of the shopping mall environment to facilitate the rehabilitation process. Through this project, focus groups were conducted with clinicians working in rehabilitation and people with disabilities and a short video was produced based on the results to sensitize clinicians about the importance of including the community setting in their practice. The CoP members were solicited to participate in the discussion groups as well as to inform the video creation in order to ensure it addressed different potential audiences. 

## 4. Discussion

This study addressed the structure, process and outcomes of a CoP using multiple data sources and perspectives. Overall, participants were active either online and/or in the meetings. The vast majority of respondents were positive about the opportunity to communicate and collaborate with others from different backgrounds and settings to discuss social participation for individuals with a disability. Participation online was quite low and was mostly used to ask questions and share information. Approximately half of the CoP members attended each in-person meeting and results indicate that participants appreciated the richness of these discussions over on-line communication. In the following section, results are discussed with respect to the current state of knowledge regarding CoPs. 

This CoP has used both online and in-person means of communication and collaboration, as do most CoPs in the healthcare sector [18]. However, results indicate great differences in the dynamics of exchanges in the online platform *versus* discussions in the meetings. As addressed in previous studies [20], facilitators play a major role in CoPs. In this case, the facilitator had to be very active in online discussions, by posting comments in attempt to increase participation of the other members, asking questions or prompting members to give their opinion on a topic, with limited success. The facilitator had to remain active throughout the 20 months and, despite the use of different strategies, the number of comments decreased over time. 

Another factor that had been identified to explain levels of sharing of knowledge using online technology is trust between members [37]. In particular, fear of losing face and being judged by other members of the group can limit a person’s inclination to participate in online discussions [38]. A recent study by Cheung *et al.* [39] also highlights the importance of feelings of reciprocity in successful online CoPs. In particular, CoP members will be inclined to continue sharing online if they encounter the reciprocity expected and if they feel they are helping others. As members in this CoP expressed through the open-ended interviews, sharing during the meetings was much easier. The animator also had to be much less active during these meetings in which participants interacted more independently. This was particularly true when they worked in smaller groups and were invited to discuss specific topics they had chosen based on their personal interest. To encourage trust and reciprocity online, as it can be observed during the in-person meetings, it would be interesting to explore strategies others have used online to encourage participation, such as holding regular virtual meetings or seminars [30,40] or separating members into online working groups with specific responsibilities [41]. Having conducted focus groups or interviews with members earlier on in the process might have helped identify these needs and preferences regarding types of activities and use of technology in the RehabMaLL CoP and enabled changes in procedures to improve levels of participation. Bezyak, *et al.* [42] have shown that conducting focus groups early on in the process can help identify specific needs of members and limits regarding the use of online technology. In particular, their participants noted that even if social media websites such as LinkedIn or Facebook are easy and convenient to use, they are often blocked in the workplace. The use of webinars to share information and videos was recommended. Results from this study are similar to recommendations regarding strategies to improve functioning of the group made by the RehabMaLL CoP members during the qualitative interviews. It is possible that conducting earlier consultations with members would have helped identify the needs, preferences and obstacles of members and facilitate their engagement, particularly online. 

Despite these different levels of participation online and in-person, the dynamics observed in this CoP seem to be consistent with the model described by Wenger and Wenger-Trayner [43], which illustrates different levels of engagement in a group. There is usually a small number of “core members”, including the facilitator, who participate actively, along with a few “active” and “occasional” members. These “occasional” members will usually participate more when the topic is of interest to them. As for the majority of members, they are often situated in what Wenger and Wenger-Trayner call the “periphery”, meaning they participate less in discussions and activities, but remain present to take in some information shared by others. These peripheral members usually feel that they have less engagement in the concerned practice. These different levels of participation can fluctuate over time for each individual, as well as for the community as a whole. These scenarios were observed in the RehabMaLL CoP. It is interesting to note that there were no clear associations between type of participant and level of engagement, with members from each type participating to different extents. 

Overall members did show appreciation for many aspects of the CoP, although they were generally not satisfied with their participation, as expressed by low results to the question “I am very satisfied with my participation in the CoP”. This question obtained only a 55.6% agreement rate. The explanation for this relatively low level of satisfaction is unclear. According to interview findings, it may be attributed to personal factors, such as dissatisfaction with their own level of engagement, in particular because of lack of time to participate, or due to some other factors. Other facets of dissatisfaction include the ease of use and function of the online platform. Future studies should examine strategies that can help overcome these common barriers in different types of CoPs. 

One of the strong points attributed to the RehabMaLL CoP by members is the diversity of its composition and sensitization to different points of views. This advantage was observed throughout the discussions and the activities, in particular for knowledge sharing across sites and backgrounds. However, this facet of the CoP might also be responsible for some of the challenges met [44]. In particular, it is usually encouraged for a CoP to have specific objectives, but that these should emerge from the participants and not be imposed [33]. In this case, the general goal of the CoP was to develop collaboration between individuals interested in social participation of people living with disabilities, in order to inform and increase uptake of research. The specific activities that emerged did help foster collaboration and did focus on social participation, as reported by our participants, but they were not exclusively oriented towards research activities. This is likely due to the diversity of participants, many of whom had little experience in research and in this type of collaboration. Given the variety of different backgrounds and settings, it has been challenging to find common projects that touch on everyone’s interest. This CoP is still in its early stages of development [16], and it is most likely that with time, members will get to know each other better, leading to greater trust and the capacity to identify clearer group objectives. Perhaps with time, the CoP will also be able to go a step further in creating new knowledge and facilitating the research process. There is a need to further understand what conditions can help increase collaboration, knowledge sharing and creation with a group as diverse as this one. As Bowen and Graham have recently pointed out: “more research is needed to determine what types of interaction are productive, under what conditions, and what their range of outcomes and impact might be” [2] (p. S5). Future research with the RehabMaLL CoP will focus on evaluating different strategies to include stakeholders in research activities, as well as varying types of online activities to include webinars and live online presentations and discussions.

### Study Limitations

Despite the growing interest in CoPs, there is still a lack of research looking at the outcomes of such initiatives and offering validated tools and frameworks for this assessment [20,25]. It remains unclear which aspects should be evaluated and how, in particular because of the innovative and changing nature of such groups. Our suggested framework, based on the Donabedian model of evaluation of care, addressed a wide range of potential issues and outcomes that were relevant. The study also triangulated different data sources to capture the different facets of the CoP. However, we did not interview those who discontinued their participation in the CoP; their insight would have been useful in understanding and addressing other challenges. Continued research in this field is essential to better develop and validate tools and frameworks to assess all aspects of CoPs. 

The evaluation focused on collaboration and outcomes of the RehabMaLL CoP, but did not address outcomes of the larger RehabMaLL project, including accessibility and use of the mall for people with disability. In that sense, it would have been interesting to include questions regarding the access to or use of the Mall space in the qualitative interviews, and get greater feedback on the topic from CoP members, in particular participants with a disability. 

## 5. Conclusions

Understanding the process, structure and outcomes of the RehabMaLL CoP can be useful to those interested in developing a similar group. The first 20 months of this CoP show positive outcomes of collaboration between members of different backgrounds interested in social participation of people living with disabilities. Results also suggest some challenges, in particular in regards to online participation. Future research is needed to better understand ways of increasing collaboration in a CoP with participants from such a wide range of different backgrounds and settings.
